# Association of bone mineral density with trichlorophenol: a population-based study

**DOI:** 10.1186/s12891-023-06323-y

**Published:** 2023-03-17

**Authors:** Zijian Yan, Xianmei Xiong, Jiasheng Tao, Sheng Wang

**Affiliations:** 1 The First Clinical Medical College, Guangzhou University of Chinese Medicine, Number12, Jichang Road, Baiyun District, Guangzhou, 510405 Guangdong Province China; 2 Department of orthopedics and Traumatology, Nantong TCM Hospital Affiliated to Nanjing University of Chinese Medicine, Number41, Jianshe Road, Nantong, 226001 Jiangsu Province China

**Keywords:** Trichlorophenol, Bone mineral density, Osteopenia, Osteoporosis, NHANES

## Abstract

**Background:**

Trichlorophenols (TCPs) are metabolites of several organochlorine chemicals, including chlorobenzene, hexachlorocyclohexane, and chlorophenoxy acid, present in air, surface water, soil, and sediment. Many studies have shown that endocrine disruptors (EDs)may contribute to decreased bone mass and the increased risk of osteoporosis. However, the relationship between TCP and bone mineral density (BMD) has not been studied yet.

**Methods:**

We conducted a cross-sectional study by using data from the 2005–2010 National Health and Nutrition Examination Survey (NHANES). TCP levels were measured in urine samples from 3385 participants and bone mineral density was obtained by dual X-ray absorptiometry (DXA) lumbar spine and femur scanning. Multiple regression analysis, stratified analysis, curve fitting analysis, and trend tests were used to assess the relationship between TCP and BMD.

**Result:**

After adjusting for confounding factors, the results of multiple regression analysis only showed that ln-2,4,5-TCP was negatively associated with BMD of lumbar spine. In stratified analyses, Male, Menstruating Female and Menopausal Female were divided into three groups for analysis. The results showed that ln-2,4,5-TCP and ln-2,4,6-TCP were not statistically associated with BMD in total femur, femoral neck, femoral tuberosity, intertrochanteric femur and lumbar spine, which was also confirmed by curve fitting analyses and trend tests.

**Conclusion:**

This study demonstrated that 2,4,5-TCP and 2,4,6-TCP in urine samples were not significantly associated with BMD in the US population. Therefore, 2,4,5-TCP and 2,4,6-TCP may not be detrimental to BMD.

## Introduction

The chlorophenols group comprises a total of five different types of chlorophenols, corresponding to their degree of chlorination: monochlorophenols (MCPs), dichlorophenols (DCPs), trichlorophenols (TCPs), tetrachlorophenols (TeCPs) and pentachlorophenols (PCP). TCPs including 2,4,5-trichlorophenol (2,4,5-TCP) and 2,4,6-trichlorophenol (2,4,6-TCP) are formed when three chlorine atoms are joined to one phenol molecule. TCPs have been widely used in herbicides, wood preservatives and fungicides, and as intermediates in various chemically synthesised pesticides, dyes and other products, and are widely present as environmental pollutants [1, 2]. TCPs are also metabolites of several organochlorine chemicals, including chlorobenzene, hexachlorocyclohexane, and chlorophenoxy acid, present in air, surface water, soil, and sediment. Therefore, the general population may be exposed to TCPs through ingestion of food and water or inhalation of air contaminated with TCPs or other organochlorine chemicals [3, 4]. Biomonitoring surveys of toxic substances are essential to determine average exposure levels of populations, to identify at-risk groups and to prevent further adverse effects [5]. National biomonitoring surveys for environmental contaminants such as TCP have been conducted in a number of countries, including the USA and some countries in Germany.

Osteoporosis is a common systemic bone disease worldwide that can lead to an increased risk of fracture [6]. Based on data from the United States Centers for Disease Control National Health and Nutrition Examination Survey (NHANES; 2005–2010), the National Osteoporosis Foundation estimates that more than 9.9 million Americans have osteoporosis and an additional 43.1 million have low bone mineral density (BMD) [7].Current treatment for osteoporosis is clinically well-established and the corresponding therapeutic effects are clear, with definite results obtained with bone-building drugs [8]. However, a clearer understanding of the risks that lead to osteoporosis and further prevention of osteoporosis could better improve quality of life and have important social benefits.

Epidemiological studies indicated that 2,4-dichlorophenol concentrations are associated with lower bone mineral density, higher osteopenia, and higher prevalence of osteoporosis in men [9]. However, the relationship between TCPs and bone mineral density has not been studied yet. Therefore, we used 2005–2010 National Health and Nutrition Examination Survey (NHANES) data to assess the association between urinary TCPs and BMD in the US population.

## Materials and methods

### Study pupulation

**Fig. 1:**
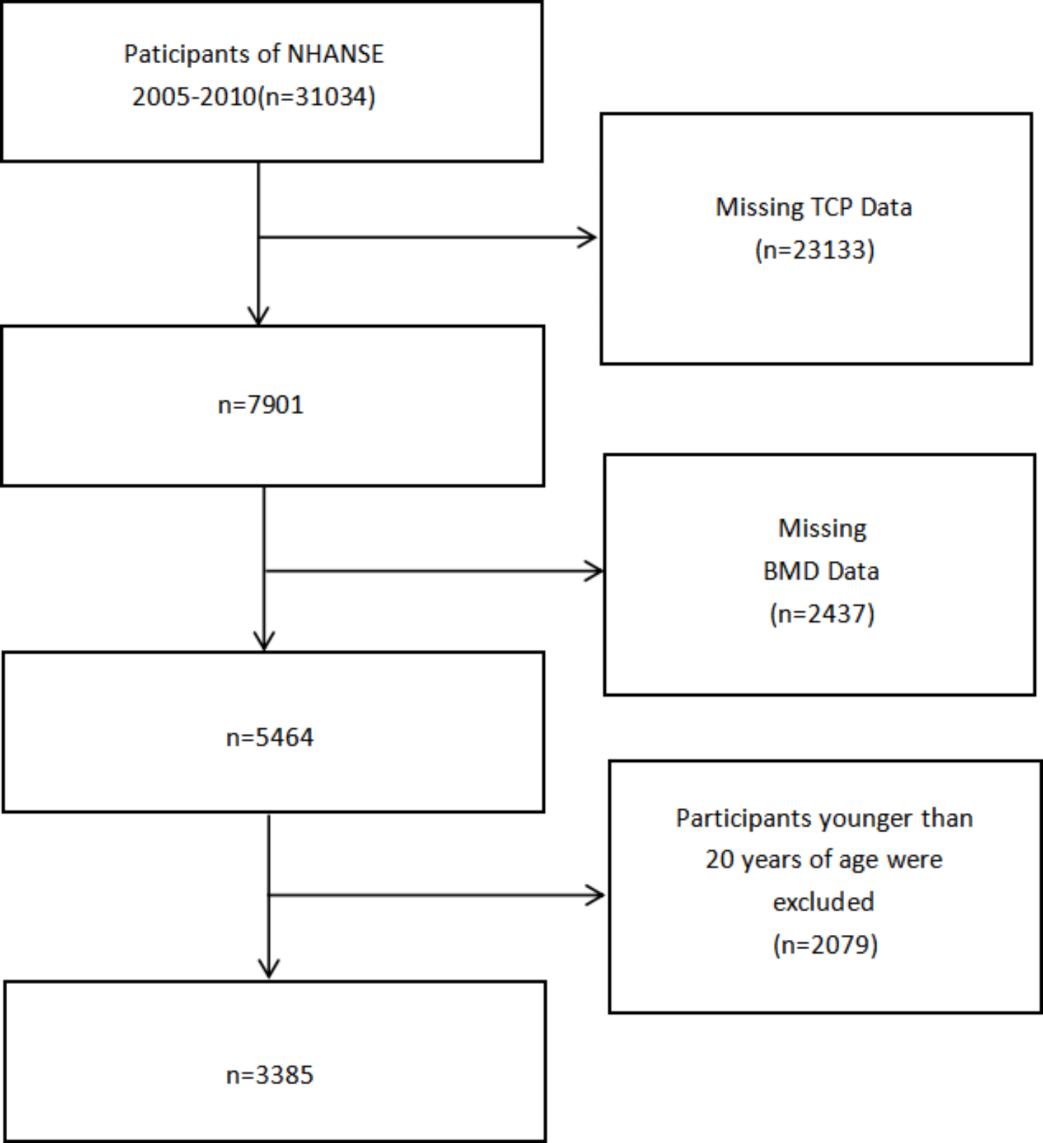
Flow chart of the screening process for the selection of eligible participants in NHANES.

The NHANES is a major program of the National Center for Health Statistics (NCHS), which is part of the Centers for Disease Control and Prevention [10]. A representative sample of non-institutional US populations is selected through a complex stratified multi-stage sampling design. The NHANES programme was approved by the Ethical Review Board of the National Center for Health Statistics of the CDC, and written informed consent was provided to all participants during the survey [11, 12]. In the three cycles of 2005–2010, 10,537, 10,149 and 10,348 people participated in the NHANES program, a total of 31,034 people as shown in Fig. 1. Our analysis was applicable to participants older than 20 years, but only those who had urine TCP and BMD information tested were combined and used for the analysis. Therefore, we first excluded participants with missing TCPs data (n = 23,133). The participants with missing BMD information was then further excluded (n = 2437). Participants younger than 20 years of age were excluded (n = 2079). Finally, this study included 3385 participants, including 1737 males and 1648 females.

### Assessment of BMD and T scores

The lumbar spine and femur scanning were acquired on Hologic Discovery model A densitometers, using software version Apex 3.2. The radiation exposure from DXA scans is extremely low at less than 20 uSv. All scans were analyzed with Hologic APEX version 4.0 software with NHANES BCA option. A high level of quality control was maintained throughout the DXA data collection and scan analysis, including a rigorous phantom scanning schedule. DXA scanning was used for eligible survey participants over the age of 8. Participants who meet one of the following criteria are prohibited from having a DXA scanning: [1] Pregnancy (positive urine pregnancy test and/or self-reported at the time of DXA examination [2]. Self-reported of radiographic contrast agent (barium) use within the past 7 days [3]. Self-reported weight over 450 pounds (209 kg) or height over 6’5” (1.96 m) [13]. The BMD of total femur, femoral neck, femoral tuberosity, intertrochanteric femur and lumbar spine were selected in this study [14]. L1-4 lumbar spine are used for lumbar spine BMD [15–17].

### Assessment of TCP

Information on urinary TCPs was obtained from the NHANES data [18]. Urine specimens are processed, stored, and shipped to the Division of Environmental Health Laboratory Sciences, National Center for Environmental Health, Centers for Disease Control and Prevention for analysis. Detailed specimen collection and processing instructions are discussed in the NHANES Laboratory/Medical Technologists Procedures Manual (LPM). According to the LPM [9], “urine samples were collected from participants with standard urine collection cups. Samples were refrigerated as soon as possible and transferred to specimen vials within 4 h of collection. At least 5 ml of urine were collected and stored frozen in borosilicate glass, polypropylene vials or specimen cups.“ Vials are stored under appropriate frozen (-20℃) conditions until they are shipped to National Center for Environmental Health for testing. A Sciex API 4000 mass spectrometer was used in negative ion APCI mode. The negative fragment ions were used for quantification of the urinary concentrations of 2,4,5-TCP and 2,4,6-TCP [19]. Creatinine-corrected urine concentrations of 2,4,5-TCP and 2,4,6-TCP were further calculated and analyzed.

### Covariates

In the NHANES database, there is a column for demographic data. In this column we collected information on the age, race, sex, body mass index (BMI), household income poverty ratio, education level, alcohol consumption, serum cotinine, milk intake, moderate physical activity, vigorous physical activity, serum calcium, serum phosphorus, serum lead and diabetes. Furthermore, we grouped women according to menstruation in 12 months, reasons for no menstruation, hysterectomy performed, and ever use female hormones as premenopausal women and postmenopausal women, respectively. Interviewer-administered questionnaires are used to record demographic information such as age, sex, race/ethnicity, household income poverty ratio, and educational attainment. Race self-reported as Mexican American, Other Hispanic, Non-Hispanic White, Non-Hispanic Black, Other. BMI was calculated by dividing body weight (in metric tons) by body height (in meters squared). Serum cotinine, calcium, lead and phosphorus were serum specimens from participants that were transported to a collaborating laboratory service for analysis. Whether participants had diabetes was considered based on diabetes self-report or fasting blood glucose or two-hour postprandial blood glucose. Vigorous physical activity was identified from the questionnaire (did you do any vigorous activities for at least 10 min that caused heavy sweating, or large increases in breathing or heart rate such as running, lap swimming, aerobics classes or fast bicycling). Moderate physical activity was determined from the questionnaire (did you do moderate activities for at least 10 min that cause only light sweating or a slight to moderate increase in breathing or heart rate such as brisk walking, bicycling for pleasure, golf, and dancing). Drinking milk was determined from the questionnaire (regular milk use 5 times per week).

### Statistical analysis

All statistical results were calculated based on NHANES sample weights. Model 1 did not adjust for any confounding factors. Model 2 was adjusted for sex, age, BMI and race. Model 3 also adjusts for sex, age, race, BMI, household income poverty ratio, education level, milk consumption, alcohol consumption, serum cotinine, serum lead, calcium concentration, phosphorus concentration, diabetes, moderate physical activity and vigorous physical activity. After adjusting for potential confounders, weighted multiple regression analysis was used to estimate the independent relationship between TCPs and BMD. Stratified analyses based on sex and menopausal state in women were then conducted to examine the association between TCPs and BMD at different sites. Finally, we proceed to the smoothing curve fitting and trend tests were performed to resolve the linear or curvilinear relationship in the subgroup analyses.

## Result

### Characteristics of the study population

The proportion of men in the study population was 51% and the proportion of women was 49%. The mean age of the study population was 46.21 years for males and 46.78 years for females, and the mean BMI was 27.86 kg/m^2 for males and 28.00 kg/m^2 for females, with no statistically significant differences in BMI. Similarly, there were no significant differences between males and females in terms of education level. The average serum calcium and serum lead in males were higher than those in females, but the serum phosphorus was lower than that in females. The percentages of smoking, alcohol consumption and milk consumption were all statistically greater for males than females. There was no statistically significant difference between males and females in moderate physical activity. However, in vigorous physical activity, the percentage of males was significantly higher than that of females. There was no significant difference in the proportion of men and women with diabetes. In total femur, femoral neck, femoral tuberosity, intertrochanteric femur and lumbar spine, males had higher mean BMD than females (Table 1).

**Table 1:** Characteristics of the study population

Variable	Male	Female	P-value
N	1737	1648	
Age(years)	46.21 ± 17.01	46.78 ± 16.93	< 0.01
Race (%)			0.23
Mexican American	351 (20.21)	308 (18.69)	
Other Hispanic	137 (7.89)	156 (9.47)	
Non-Hispanic White	838 (48.24)	772 (46.84)	
Non-Hispanic Black	326 (18.77)	339 (20.57)	
Other Race	85 (4.89)	73 (4.43)	
BMI (kg/m^2)	27.86 ± 4.97	28.00 ± 6.09	0.44
Education(%)			0.63
High School Grad or Below	468 (26.94)	432 (26.21)	
College Grad or Above	1269 (73.06)	1216 (73.79)	
Income poverty ratio(%)			0.02
≤ 0.99	325 (18.71)	360 (21.84)	
> 1	1412 (81.29)	1288 (78.16)	
Serum calcium(mg/dL)	9.49 ± 0.35	9.42 ± 0.37	< 0.01
Serum phosphorus(mg/dL)	3.65 ± 0.57	3.84 ± 0.56	< 0.01
Serum lead(ug/dL)	2.14 ± 2.14	1.46 ± 1.12	< 0.01
Serum cotinine(ng/mL)	72.61 ± 139.98	54.85 ± 124.15	< 0.01
Drink wine(%)			< 0.01
Yes	1459 (84.00)	1043 (63.29)	
No	278 (16.00)	605 (36.71)	
Drink milk(%)			< 0.01
Yes	1383 (79.62)	1206 (73.18)	
No	354 (20.38)	442 (26.82)	
Moderate physical activity(%)			0.18
Yes	802 (46.17)	723 (43.87)	
No	935 (53.83)	925 (56.13)	
Vigorous physical activity(%)			< 0.01
Yes	598 (34.43)	351 (21.30)	
No	1139 (65.57)	1297 (78.70)	
Diabetes(%)			0.1
Yes	244 (14.05)	200 (12.14)	
No	1493 (85.95)	1448 (87.86)	
Ln-2,4,5-TCP(ug/L)	-7.19 ± 0.73	-6.81 ± 0.80	< 0.01
Ln-2,4,6-TCP(ug/L)	-5.59 ± 0.71	-5.25 ± 0.75	< 0.01
BMD			
Total femur(g/cm2)	1.04 ± 0.15	0.92 ± 0.15	< 0.01
Femoral neck(g/cm2)	0.88 ± 0.14	0.82 ± 0.15	< 0.01
Femoral tuberosity(g/cm2)	0.78 ± 0.13	0.69 ± 0.12	< 0.01
Intertrochanteric femur(g/cm2)	1.22 ± 0.17	1.09 ± 0.17	< 0.01
Lumbar spine(g/cm2)	1.06 ± 0.14	1.01 ± 0.15	< 0.01

### Multiple regression analysis

The results of the multiple regression analysis are shown in Table 2. In the unadjusted model, both ln-2,4,5-TCP and ln-2,4,6-TCP were negatively correlated with BMD of total femur, femoral neck, femoral tuberosity, intertrochanteric femur and lumbar spine (P < 0.01). After adjusting for sex, age, BMI and race confounders, there was no significant linear relationship between ln-2,4,5-TCP and ln-2,4,6-TCP and BMD of total femur, femoral neck, femoral tuberosity, intertrochanteric femur. However, ln-2,4,5-TCP were still negatively correlated with BMD of lumbar spine (P = 0.02). In Model 3, ln-2,4,5-TCP were still negatively correlated with BMD of lumbar spine (P = 0.04), and not significantly correlated with BMD in other parts.

**Table 2:** Multiple regression analysis of TCP and BMD

BMD	Model 1 β (95% CI) P-value	Model 2 β (95% CI) P-value	Model 3 β (95% CI) P-value
Total femur			
ln-2,4,5-TCP	-0.056 (-0.063, -0.050) < 0.01	-0.003 (-0.009, 0.002) 0.26	-0.003 (-0.009, 0.003) 0.27
ln-2,4,6-TCP	-0.049 (-0.056, -0.042) < 0.01	-0.004 (-0.010, 0.001) 0.14	-0.005 (-0.011, 0.001) 0.11
Femoral neck			
ln-2,4,5-TCP	-0.053 (-0.059, -0.047) < 0.01	-0.001 (-0.007, 0.004) 0.70	-0.001 (-0.007, 0.004) 0.67
ln-2,4,6-TCP	-0.046 (-0.052, -0.039) < 0.01	-0.004 (-0.010, 0.001) 0.12	-0.005 (-0.010, 0.001) 0.08
Femoral tuberosity			
ln-2,4,5-TCP	-0.041 (-0.046, -0.035) < 0.01	-0.003 (-0.008, 0.002) 0.29	-0.003 (-0.008, 0.003) 0.32
ln-2,4,6-TCP	-0.037 (-0.042, -0.031) < 0.01	-0.004 (-0.009, 0.002) 0.20	-0.004 (-0.009, 0.002) 0.17
Intertrochanteric femur			
ln-2,4,5-TCP	-0.064 (-0.072, -0.057) < 0.01	-0.004 (-0.011, 0.003) 0.25	-0.004 (-0.011, 0.003) 0.26
ln-2,4,6-TCP	-0.056 (-0.064, -0.047) < 0.01	-0.004 (-0.011, 0.003) 0.23	-0.005 (-0.012, 0.002) 0.20
Lumbar spine			
ln-2,4,5-TCP	-0.033 (-0.039, -0.027) < 0.01	-0.008 (-0.014, -0.001) 0.02	**-0.007 (-0.013, -0.000) 0.04**
ln-2,4,6-TCP	-0.029 (-0.036, -0.023) < 0.01	-0.006 (-0.013, 0.000) 0.06	-0.006 (-0.013, 0.000) 0.06

Trichlorophenol: TCP; Bone mineral density: BMD; Model 1 did not adjust for any confounding factors. Model 2 was adjusted for sex, age, BMI and race. Model 3 also adjusts for sex, age, race, BMI, household income poverty ratio, education level, milk consumption, alcohol consumption, serum cotinine, serum lead, calcium concentration, phosphorus concentration, diabetes, moderate physical activity and vigorous physical activity

### Stratified analysis

In Table 3, a stratified study on the relationship between ln-2,4,5-TCP, ln-2,4,6-TCP and BMD of five study sites in men and women, as well as before and after menopause. In model 1, the results of stratified analysis showed that TCP was negatively correlated with BMD in all parts. In model 3, after adjusting for the included covariates, stratified analysis showed that ln-2,4,5-TCP and ln-2,4,6-TCP had no statistically significant relationship with BMD.

**Table 3:** Stratified analysis of TCP and BMD.

BMD	Model 1 β (95% CI) P-value	Model 2 β (95% CI) P-value	Model 3 β (95% CI) P-value
Male			
Total femur			
ln-2,4,5-TCP	-0.039 (-0.048, -0.029) < 0.01	-0.007 (-0.015, 0.002) 0.13	-0.005 (-0.013, 0.004) 0.27
ln-2,4,6-TCP	-0.028 (-0.038, -0.019) < 0.01	-0.006 (-0.014, 0.002) 0.16	-0.005 (-0.014, 0.003) 0.21
Femoral neck			
ln-2,4,5-TCP	-0.043 (-0.052, -0.034) < 0.01	-0.003 (-0.011, 0.005) 0.41	-0.002 (-0.010, 0.006) 0.62
ln-2,4,6-TCP	-0.032 (-0.042, -0.023) < 0.01	-0.005 (-0.013, 0.003) 0.19	-0.005 (-0.013, 0.003) 0.22
Femoral tuberosity			
ln-2,4,5-TCP	-0.026 (-0.034, -0.018) < 0.01	-0.006 (-0.014, 0.002) 0.13	-0.004 (-0.012, 0.003) 0.26
ln-2,4,6-TCP	-0.019 (-0.027, -0.011) < 0.01	-0.005 (-0.013, 0.003) 0.21	-0.004 (-0.012, 0.003) 0.29
Intertrochanteric femur			
ln-2,4,5-TCP	-0.045 (-0.056, -0.034) < 0.01	-0.008 (-0.018, 0.002) 0.12	-0.006 (-0.016, 0.004) 0.24
ln-2,4,6-TCP	-0.032 (-0.043, -0.021) < 0.01	-0.007 (-0.017, 0.003) 0.19	-0.006 (-0.016, 0.004) 0.24
Lumbar spine			
ln-2,4,5-TCP	-0.016 (-0.025, -0.007) < 0.01	-0.009 (-0.019, 0.000) 0.05	-0.008 (-0.017, 0.001) 0.09
ln-2,4,6-TCP	-0.013 (-0.023, -0.004) < 0.01	-0.007 (-0.016, 0.003) 0.17	-0.006 (-0.015, 0.003) 0.19
BMD			
**Menstruating Female**			
Total femur			
ln-2,4,5-TCP	-0.028 (-0.038, -0.017) < 0.01	-0.005 (-0.015, 0.005) 0.34	-0.007 (-0.017, 0.003) 0.19
ln-2,4,6-TCP	-0.031 (-0.041, -0.020) < 0.01	-0.008 (-0.019, 0.002) 0.11	-0.010 (-0.020, 0.001) 0.06
Femoral neck			
ln-2,4,5-TCP	-0.033 (-0.044, -0.023) < 0.01	-0.004 (-0.013, 0.006) 0.44	-0.005 (-0.015, 0.004) 0.28
ln-2,4,6-TCP	-0.036 (-0.047, -0.025) < 0.01	-0.007 (-0.017, 0.003) 0.15	-0.009 (-0.019, 0.001) 0.09
Femoral tuberosity			
ln-2,4,5-TCP	-0.018 (-0.027, -0.009) < 0.01	-0.004 (-0.013, 0.005) 0.39	-0.005 (-0.015, 0.004) 0.24
ln-2,4,6-TCP	-0.021 (-0.030, -0.011) < 0.01	-0.006 (-0.015, 0.003) 0.19	-0.007 (-0.017, 0.002) 0.13
Intertrochanteric femur			
ln-2,4,5-TCP	-0.030 (-0.042, -0.018) < 0.01	-0.005 (-0.017, 0.007) 0.43	-0.007 (-0.019, 0.005) 0.25
ln-2,4,6-TCP	-0.032 (-0.045, -0.020) < 0.01	-0.008 (-0.021, 0.004) 0.19	-0.010 (-0.022, 0.003) 0.12
Lumbar spine			
ln-2,4,5-TCP	-0.021 (-0.031, -0.010) < 0.01	-0.006 (-0.016, 0.005) 0.29	-0.004 (-0.015, 0.006) 0.43
ln-2,4,6-TCP	-0.024 (-0.034, -0.013) < 0.01	-0.007 (-0.018, 0.004) 0.20	-0.005 (-0.016, 0.006) 0.38
BMD			
**Menopausal Female**			
Total femur			
ln-2,4,5-TCP	-0.034 (-0.048, -0.020) < 0.01	0.004 (-0.009, 0.017) 0.53	0.005 (-0.007, 0.018) 0.41
ln-2,4,6-TCP	-0.028 (-0.044, -0.013) < 0.01	-0.000 (-0.014, 0.013) 0.96	0.003 (-0.010, 0.017) 0.60
Femoral neck			
ln-2,4,5-TCP	-0.032 (-0.045, -0.019) < 0.01	0.006 (-0.006, 0.018) 0.34	0.007 (-0.005, 0.019) 0.24
ln-2,4,6-TCP	-0.028 (-0.043, -0.014) < 0.01	-0.001 (-0.013, 0.012) 0.89	0.002 (-0.010, 0.015) 0.72
Femoral tuberosity			
ln-2,4,5-TCP	-0.022 (-0.034, -0.011) < 0.01	0.004 (-0.007, 0.016) 0.44	0.005 (-0.006, 0.017) 0.36
ln-2,4,6-TCP	-0.020 (-0.033, -0.007) < 0.01	-0.000 (-0.012, 0.012) 0.97	0.003 (-0.009, 0.014) 0.64
Intertrochanteric femur			
ln-2,4,5-TCP	-0.042 (-0.058, -0.025) < 0.01	0.003 (-0.012, 0.018) 0.71	0.004 (-0.011, 0.019) 0.58
ln-2,4,6-TCP	-0.033 (-0.051, -0.015) < 0.01	-0.000 (-0.016, 0.016) 0.97	0.004 (-0.012, 0.020) 0.60
Lumbar spine			
ln-2,4,5-TCP	-0.028 (-0.042, -0.014) < 0.01	-0.002 (-0.016, 0.012) 0.78	-0.002 (-0.015, 0.012) 0.83
ln-2,4,6-TCP	-0.024 (-0.039, -0.009) < 0.01	-0.004 (-0.018, 0.010) 0.58	-0.001 (-0.015, 0.013) 0.90

Stratified analyses based on sex and menopausal state in women

### Trend testing and curve fitting

**Fig. 2:**
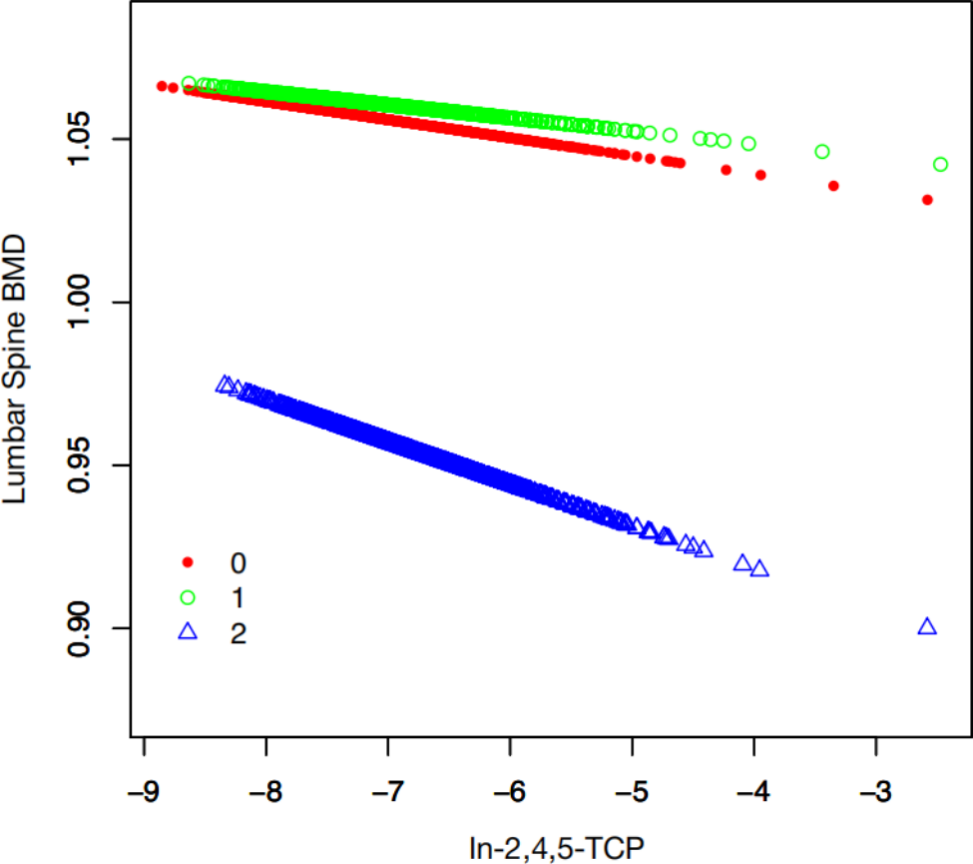
Smooth curve fit of lumbar spine BMD and ln-2,4,5-TCP. Red: Male; Green: Menstruating Female; Blue: Menopausal Female

ln-2,4,5-TCP was divided into quartiles. A curve fit analysis of BMD in the lumbar spine with ln-2,4,5-TCP is shown in Fig. 2. We found that the three lines representing male, menstruating female and menopausal female have no obvious linear relationship, so we performed a trend test to verify (Table 4). Trend test results showed that ln-2,4,5-TCP was not linearly correlated with lumbar spine BMD (P > 0.05).

**Table 4:** Trend test of lumbar spine BMD and ln-2,4,5-TCP.

BMD	Male (β (95% CI) P-value)	Menstruating Female(β (95% CI) P-value)	Menopausal Female(β (95% CI) P-value)
Q1	-	-	-
Q2	0.012 (-0.005, 0.029) 0.16	-0.017 (-0.039, 0.005) 0.14	0.001 (-0.040, 0.041) 0.98
Q3	0.002 (-0.016, 0.020) 0.85	-0.019 (-0.042, 0.004) 0.10	0.004 (-0.034, 0.042) 0.85
Q4	-0.012 (-0.032, 0.007) 0.22	-0.013 (-0.037, 0.011) 0.28	0.003 (-0.035, 0.041) 0.88

## Discussion

This is, to the best of our knowledge, the first and largest population-based study to examine the relationship between BMD and 2,4,5-TCP and 2,4,6-TCP in a nationally representative sample.

TCPs are organochlorine compounds that are found all over the environment and are known to cause cancer. Carcinogenesis bioassays were conducted by giving 2,4,6-TCP in feed to groups of 50 male and female Fischer rats and male B6C3F1 mice for two years. Dietary concentrations were 0 [20/group], 5000 [0.5%], or 10,000 [1%] ppm. It was found that TCP caused leukemia/lymphoma and liver tumor in rats [20]. 2,4,6-TCP poses a risk of human cancer, according to a number of national and international agencies. Therefore, for primary cancer prevention, exposures to 2,4,6-TCP should be minimized or eliminated, as is the case with all carcinogens that can cause cancer in animals or humans [21–25]. However, little is known about their other adverse effects on humans. Due to the numerous exposure pathways, it is difficult to measure individual exposure to TCPs. Exposure to TCPs or organochlorine chemicals that are metabolized and excreted as TCPs is indicated by TCPs excreted in the urine [2]. Urinary TCP levels can be used to accurately estimate individual exposure [26]. Organochlorine pesticides, which include 2,4,5-TCP and 2,4,6-TCP, are a large class of multipurpose chlorinated hydrocarbon chemicals that accumulate in fatty tissue and slowly degrade in the environment. Xu found that exposure to TCP may increase the risk of behavioural impairment in children in a 1999–2004 NHANES study of 2546 children [27]. Several biomonitoring studies on phenolic compounds have shown that the determination of the actual urinary excretion would provide a reliable estimation of individual exposure and that the levels of chemicals excreted from the body vary significantly depending on the population studied, reflecting differences in race and ethnicity [28–30]. In comparison to concentrations found in the United States and Canada, TCP exposure is still fairly common, but exposure levels among children and adolescents in Germany were generally low [31]. Geometric mean urinary 2,4,5-TCP concentrations decreased with age and increased with education level and income. Age remained significantly related to urinary 2,4,5-TCP concentration after the adjustment of covariates [32]. Therefore, in this study, race, age, education level, and income were considered for adjustment as covariates. A Korean study of 165 girls aged 7 to 8 years found that chlorophenol exposure was positively associated with central obesity in Korean girls [33]. While it is common knowledge that weight gain builds bone, several obesity-related mechanisms make bone more fragile. These include an increase in inflammatory cytokines that activates bone-resorbing osteoclasts, mutations in the FTO gene, increased osteoblast senescence caused by obesity, and an increased production of bone marrow fat cells at the expense of bone-forming osteoblasts [34]. Therefore, the covariate also includes BMI.

Although the aforementioned metabolites are considered to be endocrine disruptors (EDs), only a small number of population-based studies have examined the relationships between these metabolites and osteoporosis, which is another important endocrine disorder. One recent study primarily on personal care products found that in men and premenopausal women, higher paraben concentrations, particularly ethyl-, methyl-, and propylparabens, were linked to higher BMD in the femoral neck, intertrochanter, and lumbar spine. Men had a higher prevalence of osteopenia/osteoporosis and a lower BMD when exposed to 2,4-dichlorophenol. Postmenopausal women were found to have a higher prevalence of osteopenia and osteoporosis in the lumbar spine when exposed to bisphenol A. Men and premenopausal women tended to have a higher BMD of the femur when exposed to benzophenone-3 [9]. However, to date, no studies have shown whether TCP affects bone health in humans. Only one animal study showed that development of zebrafish head cartilages was seriously affected by exposure of triclosan, 2,4-dichlorophenol and 2,4,6-trichlorophenol [35].

A total of 3385 participants were included in this study, including 1737 males and 1648 females. The mean ln-2,4,5-TCP and ln-2,4,6-TCP for males were − 7.19 ug/L and − 5.59 ug/L, respectively. The mean ln-2,4,5-TCP and ln-2,4,6-TCP for females were − 6.81 ug/L and − 5.25 ug/L. Therefore, males have less ln-TCP on average than females. This may be related to the difference in the metabolic capacity of men and women. In the unadjusted model, both ln-2,4,5-TCP and ln-2,4,6-TCP were negatively correlated with BMD of five study sites. However, after adjusting for confounders such as sex, age, race, BMI, household income poverty ratio, education level, milk consumption, alcohol consumption, serum cotinine, serum lead, calcium concentration, phosphorus concentration, diabetes, moderate physical activity and vigorous physical activity, only ln-2,4,5-TCP were still negatively correlated with BMD of lumbar spine. The results of stratified analysis showed that there was no significant linear relationship between ln-2, 4, 5-TCP and BMD of lumbar spine in the three stratifications of male, menstruating female and menopausal female. Curve fitting analysis and trend test also verified this conclusion. Therefore, ln-2,4,5-TCP and ln-2,4,6-TCP levels in the US population may not adversely affect BMD. However, this result is only applicable to the American population. Due to the differences in the production, lifestyle and ethnicity of each country, more research may be needed to evaluate the harm of TCP.

This study had a number of limitations. First, this study was a cross-sectional one. As a result, it is impossible to fully verify causal relationships or coincidental phenomena. Second, it is impossible to completely rule out residual and unmeasured confounding factors in this observational study. Thirdly, additional research is required to ascertain the underlying mechanism and direction of the associations’ causality. Last but not least, since this paper is the first study to study the relationship between TCP and human bone health, there are not many literatures that can be cited or referenced. However, this is also a major innovation of this study.

### Conclusion

This study demonstrated that 2,4,5-TCP and 2,4,6-TCP in urine samples were not significantly associated with BMD in the US population. Therefore, 2,4,5-TCP and 2,4,6-TCP may not be detrimental to BMD.

## Data Availability

The data for this study are all available in NHANES (https://www.cdc.gov/nchs/nhanes/index.htm).
